# Metabolic and Nutritional Issues after Lower Digestive Tract Surgery: The Important Role of the Dietitian in a Multidisciplinary Setting

**DOI:** 10.3390/nu16020246

**Published:** 2024-01-12

**Authors:** Alejandra Utrilla Fornals, Cristian Costas-Batlle, Sophie Medlin, Elisa Menjón-Lajusticia, Julia Cisneros-González, Patricia Saura-Carmona, Miguel A. Montoro-Huguet

**Affiliations:** 1 Department of Digestive Surgery, University Hospital Miguel Servet, 50009 Zaragoza, Spain; 2 Department of Nutrition and Dietetics, Bradford Teaching Hospitals NHS Foundation Trust, Bradford BD9 6RJ, UK; cristian.costas2@bthft.nhs.uk; 3 City Dietitians, London WC2E 7PP, UK; sophie@citydietitians.co.uk; 4 Gastroenterology, Hepatology and Nutrition Unit, University Hospital San Jorge, 22004 Huesca, Spain; emnjn08@gmail.com; 5 Faculty of Health and Sport Sciences, University of Zaragoza, 22002 Huesca, Spain; 683804@unizar.es (J.C.-G.); patriciasaura.ps@gmail.com (P.S.-C.); 6 Department of Medicine, Faculty of Health and Sport Sciences, University of Zaragoza, 22002 Huesca, Spain; 7 Aragon Health Research Institute (IIS Aragon), University of Zaragoza, 22002 Huesca, Spain

**Keywords:** malnutrition, metabolism, micronutrients, nutritional deficiencies, nutrition assessment, dietitian, colectomy, health costs, low anterior resection, abdominoperineal resection, proctocolectomy, IBD, short bowel syndrome, intestinal failure (IF), D-lactic acidosis, refeeding syndrome, IF-associated liver disease, home parenteral nutrition, enteral nutrition, intestinal transplantation

## Abstract

Many patients undergo small bowel and colon surgery for reasons related to malignancy, inflammatory bowel disease (IBD), mesenteric ischemia, and other benign conditions, including post-operative adhesions, hernias, trauma, volvulus, or diverticula. Some patients arrive in the operating theatre severely malnourished due to an underlying disease, while others develop complications (e.g., anastomotic leaks, abscesses, or strictures) that induce a systemic inflammatory response that can increase their energy and protein requirements. Finally, anatomical and functional changes resulting from surgery can affect either nutritional status due to malabsorption or nutritional support (NS) pathways. The dietitian providing NS to these patients needs to understand the pathophysiology underlying these sequelae and collaborate with other professionals, including surgeons, internists, nurses, and pharmacists. The aim of this review is to provide an overview of the nutritional and metabolic consequences of different types of lower gastrointestinal surgery and the role of the dietitian in providing comprehensive patient care. This article reviews the effects of small bowel resection on macronutrient and micronutrient absorption, the effects of colectomies (e.g., ileocolectomy, low anterior resection, abdominoperineal resection, and proctocolectomy) that require special dietary considerations, nutritional considerations specific to ostomized patients, and clinical practice guidelines for caregivers of patients who have undergone a surgery for local and systemic complications of IBD. Finally, we highlight the valuable contribution of the dietitian in the challenging management of short bowel syndrome and intestinal failure.

## 1. Introduction

Dietitians’ involvement in healthcare is growing, and the implementation of their knowledge is of considerable value. Some of the subjects that can benefit from the knowledge of the registered dietitian (RD) include sports, eating disorders, oncology patients, geriatric patients, critically ill patients, and a vast miscellany of digestive diseases that cause numerous nutritional and metabolic complications. Digestive surgery causes the development of the relevant anatomical and functional changes that directly impact these patients’ metabolic and nutritional state.

The list of abdominal surgical operations that impact on the nutritional status of patients is wide. It includes a miscellany of operations affecting the upper and lower digestive tracts. The first ones include the resection and replacement of the esophagus, different types of gastrectomy, bariatric surgery (e.g., Roux-en-Y gastric bypass or biliopancreatic bypass with duodenal switch), cholecystectomy, and pancreaticoduodenectomy. Its nutritional consequences are not the subject of this review, which has been limited to the analysis of lower digestive tract surgery.

The most common indications for lower gastrointestinal (GI) surgery (intestinal resection, colectomy, and stomas) tend to be malignancy, inflammatory bowel disease (IBD), and some benign pathologies, such as intestinal occlusion due to post-surgical adhesions, hernias of the abdominal wall or internal hernias, volvulus, and, finally, the complications of diverticular disease (abscesses, fistulas, strictures, and perforation). Other injuries, such as intestinal ischemia and trauma, can have devastating consequences due to the extent of the resection and the intense systemic inflammatory response that generates metabolic stress and malnutrition.

The role of the RD in this field is exciting and encompasses a wide range of scenarios with variable complexity. Among them, it is worth highlighting: (1) the effects of small bowel resection on the absorption of macronutrients and micronutrients that differs according to the location and size of the anatomical surface affected by the resection; (2) the impact of a colectomy (ileocecectomy, low anterior resection, abdominoperineal resection, and proctocolectomy), which requires specific dietary and nutritional advice, especially concerning anterior abdominal resection syndrome or the water and electrolyte losses associated with proctocolectomy; (3) considerations related to both caloric needs and macronutrient and/or micronutrient requirements (specifically vitamin B12, zinc, vitamin D, and iron) in ostomy patients; (4) screening, evaluation, and nutritional support for patients with IBD, including peri-operative nutritional support, the management of specific scenarios resulting from local complications (e.g., fistulas, abscesses, and strictures), or the need for bowel resection, especially if it ends up being extensive, affects the terminal ileum, or involves the ileocecal valve; and (5) finally, an experienced dietitian’s involvement is essential to help manage short bowel syndrome (SBS), a severe disorder with both short- and long-term repercussions for patients. Its prevalence has doubled over the past 40 years, and its nutritional management is a significant challenge that requires the collaboration of an interdisciplinary team. 

This article provides a comprehensive overview of the nutritional and metabolic consequences of different types of lower GI surgery, as well as the role of the RD in the setting of comprehensive patient healthcare. The present article has tried to send messages in two directions: (1) it is important that the clinician has the support of an experienced dietitian to improve the outcomes of patients undergoing a digestive surgery, and (2) it is equally essential that the dietitian knows the pathophysiologic mechanisms responsible for the symptoms to acquire the competencies and skills and also the authority necessary to justify his or her intervention in the team. Surgeons, dietitians, nurses, and gastroenterologists with a global view of this disease have been involved in drafting this manuscript.

## 2. Small Bowel Surgery

The most common indications for small bowel resection in adults include Crohn’s disease-related complications, occlusions due to post-surgical scarring, hernias of the abdominal wall, malignancy, trauma, radiation, and mesenteric ischemia. A period of starvation (“nil per os”) is common practice after a gastrointestinal surgery, during which an intestinal anastomosis has been formed. The purpose of *nil per os* is to prevent post-operative nausea and vomiting and to protect the anastomosis, allowing it time to heal before being subjected to food stress. However, the accumulated evidence from different clinical and experimental studies strongly suggests that the early initiation of feeding has more advantages than disadvantages [1,2,3]. Post-operative dysmotility predominantly affects the stomach and colon, as it is assumed that normal small bowel function recovers within 4 to 8 h after a laparotomy, so that feeding in the following 24 h should be well tolerated and compatible with the proper assimilation of nutrients. In addition, severe malnutrition and starvation decrease the production of collagen tissue necessary for healing at the anastomosis, resulting in sub-optimal scar quality. In contrast, an early supply stimulates the growth of the villi and promotes the deposition of collagen where it is most needed. The impact of sarcopenia in various types of surgery and the loss of muscle mass after a surgery constitute a risk that must be considered [4].

As fluid reabsorption occurs at the villus cells of the small and large intestines, a wide resection of either one could lead to fluid and electrolyte disturbances. Also, after a wide resection of the small bowel, a malabsorption of vitamins or minerals (such as vitamins A and D, folate, calcium, magnesium, and iron) could occur. In some cases, the intestinal dysfunction that occurs after an extensive bowel resection is so severe that it leads to intestinal failure with clinical and metabolic consequences that make artificial nutritional support necessary. 

**Figure 1:**
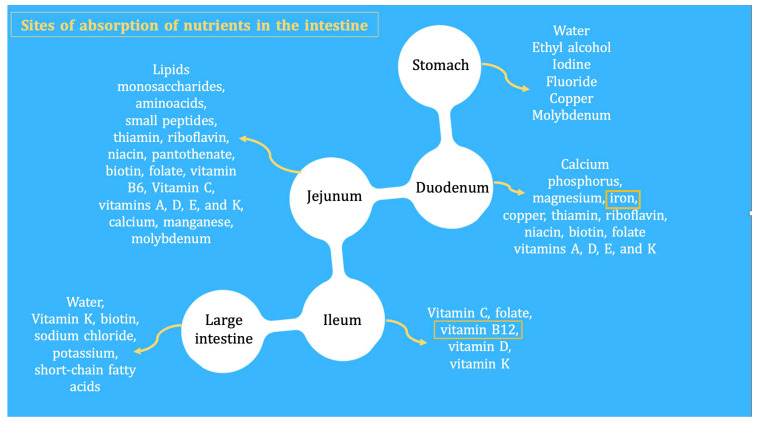
Patients who have undergone an intestinal resection may suffer from deficiencies of micronutrients/vitamins and minerals (which, in small proportions, are essential to preserve life). For this reason, these patients should be followed and monitored long term for deficiencies of iron, albumin, vitamin B12, erythrocyte folate, vitamin D3, biotin, thiamine, riboflavin, copper, selenium, zinc, and magnesium. The levels of fat-soluble vitamins may be altered due to alterations in transporter proteins, which, like albumin, decrease in systemic inflammatory states. Yellow frames indicate sites of absorption of nutrients in the intestine.

Figure 1 shows the anatomical locations where macronutrients and micronutrients are absorbed in the intestine and the potential nutritional consequences of lower GI surgery. After a bowel resection, some patients develop spontaneous intestinal adaptations and hyperphagia. Following an intestinal resection, many patients initiate a process of intestinal adaptation driven by polyphagia itself, which contributes to promoting morphological changes such as the growth and elongation of villi and microvilli, the proliferation (hyperplasia) of crypts, and a progressively larger diameter of the intestinal lumen. This phenomenon attenuates the effects of macronutrient and micronutrient malabsorption in the long term (see below) [5].

## 3. Colectomy

The colon has a vital role in the absorption of water, electrolytes, and short-chain fatty acids. Consequently, a colonic resection can cause bowel function changes, which can significantly affect patients’ quality of life. Although follow-ups focus on detecting recurrent cancer, many patients struggle with challenging bowel function after treatment, yet few patients are referred for specialist help. Loose stool, increased bowel frequency, and/or nocturnal defecation following right-sided colectomy occurs in approximately one in five patients. Bowel adaptation following surgery occurs in the first 2 years in adults, and some of these symptoms may improve spontaneously with time [6,7]. In a similar way to enterectomy, there are several scenarios in which colectomy involves metabolic and nutritional sequelae (Figure 1).

### 3.1. Ileocecectomy

The active and passive absorption of bile acids in the distal ileum allows for the reabsorption of 95% of those acids back into the bloodstream (and, therefore, to the liver). In comparison, the remaining 5% is eliminated through feces. Resection of the terminal ileum and ileocecal valve increases the amount of secondary bile acids (mainly deoxycholic acid) that reach the colon. The presence of these acids at the colic level stimulates the secretion of water and electrolytes, inhibiting sodium reabsorption, increasing intestinal motility, and shortening colonic transit time, which leads to diarrhea, abdominal distension, urgency, and fecal incontinence. This clinical picture, mainly distinguished by chronic diarrhea, was described by Hofmann in 1967 and called choleretic enteropathy [8]. This is currently termed bile acid malabsorption type one, caused by the failure of the active transport of bile acids after an ileal resection, disease, or bypass of the terminal ileum [9]. The mainstay of treatment for chronic bile acid diarrhea has been increased water and electrolyte (e.g., potassium and sodium) intake and the administration of ion-exchange resin (e.g., cholestyramine or colesevelam). After starting treatment with bile acid sequestrants, patients report less frequent and more solid stools, leading to the disappearance of incontinence and fecal urgency. However, constipation, abdominal distension, cramps, or nausea may appear, requiring dose adjustment until finding what is best tolerated [10,11]. As bile acid sequestrants can bind to other compounds, chronic treatment with cholestyramine may lead to severe deficiencies in fat-soluble vitamins (e.g., vitamins A, D, E, and K), so periodic measurement is suggested [11].

### 3.2. Low Anterior Resection and Abdominoperineal Resection

Patients with resectable rectal cancer undergo one of two operations: a low anterior resection (LAR) or an abdominoperineal resection. In the first case, it is frequent to create a temporary diverting loop ileostomy, while in the second case, an end colostomy is mandatory. 

Diversion colitis is the chronic inflammation of a dysfunctionalized segment of the colon following the performance of a temporary stoma. This form of inflammation is associated with a failure in the synthesis of short-chain fatty acids (SCFAs) and other luminal nutrients that stimulate colonocyte growth and proliferation, as well as changes in the microbiota flora, endoscopic alterations, histological changes, and abnormal serum inflammatory markers [12,13]. Most patients with diversion colitis are asymptomatic, but in a small proportion of patients, symptoms can significantly impact the quality of life. The most common symptoms in adults are tenesmus, urgency, bloody and/or mucus discharge, and abdominal pain [3,11]. In rare cases, patients have severe bleeding that requires transfusions, diarrhea, or sepsis from deep ulceration, protein-losing colopathy, and malnutrition [14]. Patients who are not candidates for the restoration of intestinal continuity may be treated with short-chain fatty acid enemas, topical 5-aminosalicylic acid agents, topical glucocorticoids, and proctectomy or sigmoid colectomy with proctectomy for refractory symptoms [15,16].

Today, a notable proportion of patients with rectal cancer eligible for radical surgery undergo a procedure leading to the preservation of the anal sphincter. LAR with total mesorectal excision will cause a loss of the reservoir’s function, including a reduction in storing as well as a markedly disturbed evacuation, thus leading to a significant impairment of rectal compliance. These disorders, together with other alterations caused by neoadjuvant therapy (e.g., radiation therapy), result in a combination of symptoms summarized under the term “low anterior resection syndrome” (LARS) [17,18], which, individually or in combination, can lead to a detrimental effect on patients’ quality of life (QOL) [19] These symptoms include urgent defecation, increased stool frequency, unproductive defecation, repeated painful stools, emptying difficulties, soiling, and, eventually, incontinence [17,20]. Dietary modification is regarded as the first-line therapy for patients suffering from LARS-mediated symptoms. These dietary suggestions are summarized in Table 1 [21,22,23]. Other therapies include medications that slow transit and improve external sphincter tone (e.g., loperamide), pelvic floor rehabilitation, consisting of muscle exercise techniques as well as biofeedback training, transanal irrigation, and neuromodulation [17].

### 3.3. Proctocolectomy

Surgery in the form of a proctocolectomy allows for patients with severe refractory ulcerative colitis to be rescued. Often, these patients arrive in the operating room in deleterious nutritional conditions due to protein-losing colopathy, anemia, and metabolic stress associated with the systemic inflammatory response. This complex scenario requires the appropriate peri-operative nutritional support to optimize outcomes. 

In healthy adults, approximately 1 L to 1.5 L of fluid enters the colon each day, and all but 150 mL is reabsorbed. Thus, following a total colectomy, it is common to have a high level of intestinal fluid loss and metabolic derangement. During the acute stage, intravenous fluid replacement with normal saline (0.9%) and supplemental potassium and magnesium are important. The oral intake should be adequate to compensate for all the losses and to maintain a urine output of at least 1 L/day. Hypertonic fluids (e.g., fruit juices) should be avoided, as they contribute to osmotic diarrhea. These dietary measures can be supplemented with motility-slowing agents (e.g., loperamide), balancing the risk–benefit of their potential effects on bacterial sequestration in the small intestine. In the long term, adaptation is highly variable and usually occurs during the first two years following a proctocolectomy in adults. This process includes structural changes (e.g., increased protein and DNA content and villus lengthening) and functional changes, including brush border membrane enzymatic activity modifications [24,25,26].

In addition to absorbing fluid, the colon can absorb some nutrients, primarily in the form of fermented malabsorbed carbohydrates. In healthy adults, the colon absorbs up to 15 percent of their daily energy requirements. Therefore, the loss of the colon not only involves a loss of fluids and electrolytes but also of energy. In the absence of the colon, a diet rich in simple carbohydrates can be disadvantageous because concentrated carbohydrates have a high level of osmolarity, which can lead to diarrhea. Complex carbohydrates are preferred, and simple sugars should generally be limited. Lactose should not be restricted unless the patient is clearly lactose intolerant, given it is an important source of calcium and calories.

## 4. Stoma Formation

**Figure 2:**
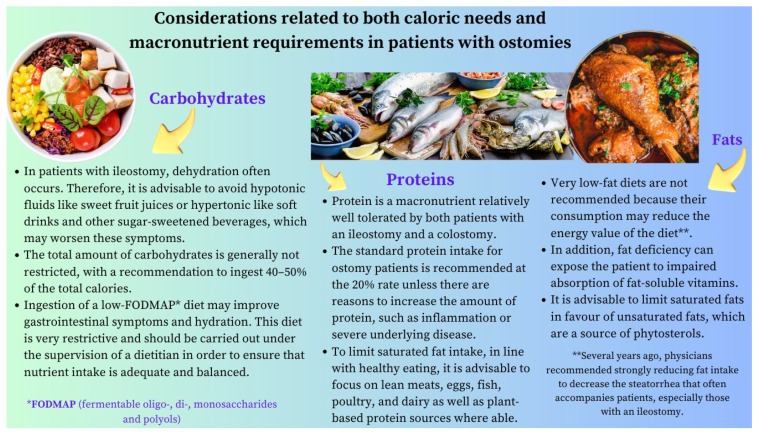
Dietary advice for ostomy patients. This figure summarizes the key considerations that are most likely to improve the nutritional status, symptoms, and quality of life in these patients. It is worth emphasizing that the nutritional status of these individuals also depends on their underlying disease state (e.g., IBD or a malignancy) characterized by periods of remission and exacerbation. Thus, the best approach for the comprehensive care of a patient with an ostomy is to provide a multidisciplinary team, including a dietitian with ample expertise in surgical nutrition.

An ileostomy or colostomy creation may be required temporarily or permanently for the management of a variety of pathologic conditions. Surgeons use various techniques to divert gastrointestinal contents, which helps avoid intra-abdominal contamination and preserve/safeguard anastomoses if continuity can be restored [27]. A GI surgery resulting in stoma formation can pose risks for patients as a result of the potential for pre-operative malnutrition caused by the underlying disease and prolonged periods of fasting during the immediate pre- and post-operative periods. Concerns over diet and nutrition are common among ostomists and their carers. Nutritional complications are usually caused by stoma outputs, the stoma site, and preexisting diseases (Figure 2) [27,28,29,30].

### 4.1. Ileostomy

The average output of an ileostomy patient ranges from 500 mL to 1300 mL a day, with a significant amount of sodium and potassium. During the early post-operative period and episodes of gastroenteritis, the daily output can be 1800 mL or even higher, favoring dehydration [28]. In fact, dehydration is the most common cause for hospital readmission after an ileostomy surgery [29,30,31,32]. 

Patients with an ileostomy should be counseled to increase their daily fluid intake above the recommended for the general population by at least 500 mL to 750 mL and to drink even more during periods of high-volume output or profuse sweating. Patients should be advised that certain sports drinks may even exacerbate stoma output and dehydration, and that the use of oral rehydration solutions with adequate concentrations of sodium and glucose are preferable. Other fluids whose composition is suitable in this context are water, broths, and vegetable juices. The first-line management of patients with an elevated ileostomy output (defined as >1.5 L/day) should include gel-forming fiber supplementation (e.g., psyllium husk), which can slow the transit time by absorbing water and forming a gel-like consistency [33,34,35,36]. It is recommended to escalate the dose gradually, up to four times a day, until the transit time is decelerated [37]. 

Patients who do not respond to this treatment may benefit from the use of antimotility agents (e.g., loperamide, one tablet two to three times a day based on their stoma output). However, using various antimotility agents at the same time (e.g., tincture of opium) can lead to paralytic ileus [38,39].

Another issue with dietary implications for patients with an ileostomy is the production of gases from carbohydrate fermentation. Therefore, choices of food can influence the amount of gas and the consistency and odor of the effluent [40,41]. Patients should be aware that the usual “time lag” between the ingestion of a gas-producing food and the development of flatulence ranges from two to four hours in the case of an ileostomy and from six to eight hours in the case of a distal colostomy. 

A dietitian can provide valuable support by informing these patients about some foods containing raffinose, a trisaccharide that is composed of glucose, fructose, and galactose that is fermented by bacteria in the intestinal lumen and enhances gas production (Table 2) [42,43,44]. 

In a study of 783 participants living with an ileostomy, 17% had iron deficiency anemia, 31% were deficient in vitamin B12, 13% had a vitamin D deficiency, and 8% were deficient in zinc. Therefore, protocols to include the monitoring of micronutrients in people with an ileostomy are essential, as is educating patients around the signs and symptoms of common nutrient deficiencies [45].

### 4.2. Colostomy

**Figure 3:**
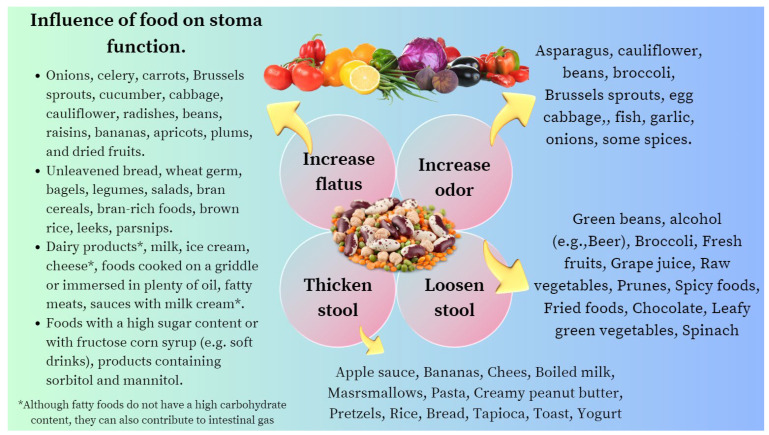
List of foods that contribute to the deterioration of the health-related quality of life of ostomized patients by causing an unpleasant odor, irritation of the peristomal skin, or increased output of fluid through the stoma.

Colostomy patients should be encouraged to ingest sufficient fiber (20 g/day to 35 g/day) and fluids (at least 1.5 L to 2 L/day) to prevent constipation and should also be counseled regarding gas-producing foods and lag time (Figure 3). If constipation does occur, it can be managed with laxatives, stoma disimpaction, or colonic irrigation. The patient should also be informed that intermittent mucoid discharge is normal either from the distal ostomy site (mucus fistula) or from the anus with ostomies that have a defunctionalized distal limb [27]. Finally, a balanced diet and adequately conducted nutritional education play a key role in preventing peristomal complications and deficiencies of any nutrients. In summary, the best approach for the comprehensive care of a patient with an ostomy is to provide a multidisciplinary team: a doctor, an ostomy nurse, a psychologist, and a dietitian [46,47,48,49].

## 5. IBD Surgery

**Figure 4:**
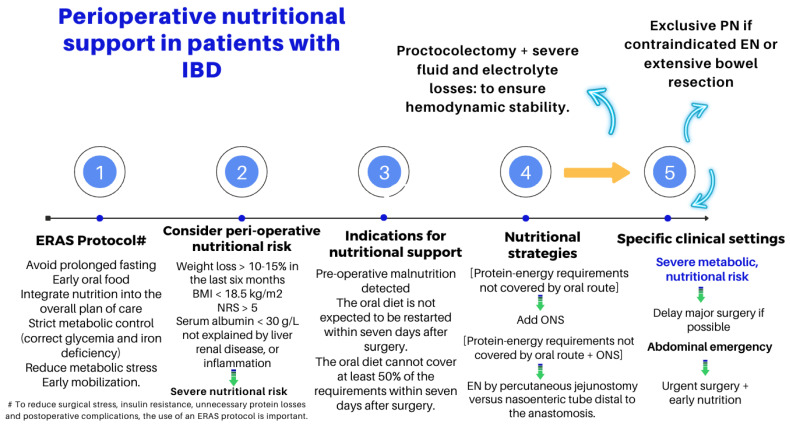
Key points in the peri-operative nutritional management of patients with IBD [50].

ESPEN renewed, in 2023, the clinical practice guidelines on the nutritional management of IBD by formulating up to 71 recommendations based on the best scientific evidence available [50]. The impact of IBD on nutritional status can be severe, ranging from generalized weight loss and growth failure to deficiencies in specific vitamins and trace elements [51]. Table 3 summarizes the reasons for malnutrition in IBD, and Figure 4 outlines some of the critical issues in the nutritional assessment and management of the patients undergoing a surgery due to experiencing local or systemic complications of their IBD.

## 6. Short Bowel Syndrome (SBS)

Short bowel syndrome (SBS) is a malabsorptive condition that is most often caused by a massive resection of the small intestine [52]. Its prevalence is 3–4 per million [53] and occurs in about 15% of adult patients undergoing an intestinal resection, either massive (3/4) or from multiple sequential resections (1/4) [54]. Although its causes may be diverse, in the present manuscript, we will refer to the one that results from a massive bowel resection [55]. SBS and intestinal failure (IF) are not necessarily synonymous. Intestinal failure describes the state when an individual’s gastrointestinal function is inadequate to maintain his or her nutrient and hydration status without intravenous or enteral supplementation [56,57,58]. Therefore, although SBS is probably the most frequent cause of IF, a significant proportion of patients with SBS will achieve intestinal autonomy and thereby avoid lifelong parenteral nutritional (PN) support. A detailed description of the pathophysiology, clinical presentation, complications, and medical and surgical management of this entity is beyond the scope of this overview, but we will highlight some aspects that emphasize the relevance of interdisciplinary handling and the role of experienced dietitians in the complex management of this clinical condition.

The loss of substantial areas of absorption associated with bowel resection diminishes the contact time with the mucosa, leading to malabsorption and diarrhea. However, the pathogenesis of diarrhea in SBS is influenced by other possible factors (Table 4). The magnitude and nature of the nutritional and metabolic complications of SBS depend on several factors, including [58]:

(1) Loss of absorptive surface area and, crucially, the length of the remaining intestine.
(2) Status and functional capacity of the intestinal mucosa.
(3) Loss of site-specific transport processes.
(4) Loss of site-specific endocrine cells and gastrointestinal (GI) hormones.
(5) Loss of the ileocecal valve (Figure 3).

In addition to all of these factors, not only the amount and specific location of the removed intestine but also the formation of a stoma, or alternatively an anastomosis, must be considered. If a jejunoileal resection is performed, with a remaining jejunum under 50 cm, the patient will experience diarrhea, steatorrhea, and progressive weight loss. Thus, the risk of requiring long-term parenteral nutrition (PN) is very high. However, in the rare event that a jejunal resection can be performed while preserving more than 10 cm of terminal ileum, it would likely not lead to undernutrition, and so these patients would probably not require long-term PN support. On the other hand, jejunoileal resection, colectomy, and the formation of a stoma (end jejunostomy) are presented as the most deleterious options, leading to dehydration, electrolyte disturbance, hemodynamic instability, and a high risk of renal failure immediately after surgery due to large stomal water and sodium losses [53,55,57].

### 6.1. Clinical Presentation

#### 6.1.1. Global Outlook

**Figure 5:**
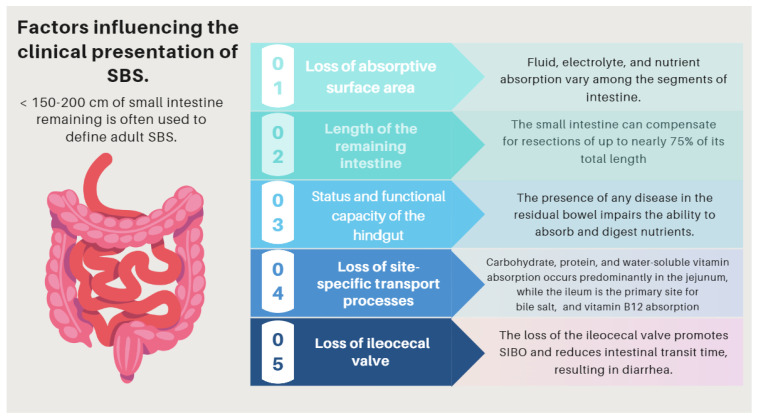
Factors influencing the pathophysiology of SBS. SBS: short bowel syndrome.

The clinical presentation of SBS comprises a range of intestinal and extraintestinal manifestations (see related complications). It may be categorized into three symptom patterns: First, those that depend on the impairment of absorption and/or stimulation of water and electrolyte secretion. Diarrhea, dehydration, and undernutrition are the most prominent symptoms in this group, and their severity mainly depends on the factors listed above (Table 4, and Figure 5) and especially on the location and extent of the removed intestinal segment. Thus, some functions of the ileum (e.g., bile salt and vitamin B12 absorption) are specific to this anatomical segment and cannot be supplied by the jejunum [59,60,61,62,63]. In addition, the ileum typically reabsorbs a large portion of the fluid secreted by the jejunum. Therefore, patients who lose a significant portion of the ileum have a limited capacity to absorb fluids and electrolytes and have difficulties tolerating high-osmolarity liquids [64]. On the other hand, losing the ileocecal valve increases the risk of SIBO and enteropathy, resulting in multiple intestinal symptoms that depend on dysbiosis, like bloating, diarrhea, and abdominal discomfort [65,66]. 

Second, those reflecting specific metabolic disturbances or inherent to the specific nutritional support these patients receive and manifested by extraintestinal symptoms and signs (e.g., refeeding syndrome, D-lactic acidosis, IF-associated liver dysfunction, biliary gallstones, or nephrolithiasis [67,68,69,70,71,72,73,74] (Table 5).

Third, those dependent on the insertion of central catheters (e.g., sepsis or central line thrombosis) which are necessary for PN support (Table 5).

**Table 5:** A list of nutritional and metabolic consequences that are related to the pathophysiology of SBS.

Complications Related to SBS	Pathogenesis
(I) Complications related to SBS pathophysiology and its underlying pathology	The pattern of nutrient absorption native to the parts of the gastrointestinal tract is shown in Figure 1.
Peptic ulcer	Hypergastrinemia resulting from a failure of enterogastrone release (e.g., VIP, GIP, neurotensin, peptide YY, and GLP-1). Treatment with antisecretory drugs could also aggravate SIBO due to hipoclorhydria [73].
Electrolyte disturbances: hypocalcemia, hypokalemia, and hypomagnesemia	Occur especially when large-volume diarrhea is present. (e.g., associated with an end jejunostomy).
D-lactic acidosis (D-LA)	The SBS microbiota, since it is rich in Lactobacillus, leads to the accumulation of fecal lactate. Lactate does not accumulate in healthy human feces because it is absorbed by intestinal cells, but in some SBS patients, the high amount of lactate found in feces indicates that production exceeds absorption capacities by the host. Excess lactate released into the colon is fermented by bacteria and converted into D-lactate, which has neuro-toxic effects [67,68,69].
Cholelithiasis	In the presence of an ileum resection, it breaks the enterohepatic circle of bile salts, causing a reduced biliary excretion and a marked decrease of the bile salt pool in the duodenal lumen. Consequently, cholesterol is oversaturated, favoring the formation of biliary stones [70,71].
Nephrolithiasis	As a result of steatorrhea, increased free fatty acids are available to bind to calcium, resulting in an increased concentration of non-bound oxalate, which is easily absorbed across the colonic mucosa, where it is moving to the kidneys. Nephrolithiasis is more common among patients with an intact colon. The risk of nephrolithiasis is enhanced by volume depletion, metabolic acidosis, and hypomagnesemia, resulting in decreased renal perfusion, urine output, pH, and citrate excretion [72,73].
Metabolic osteopathy	Metabolic changes that occur in SBS result in the depletion of calcium, magnesium, and vitamin D, which results in the demineralization of bone. The release of pro-inflammatory cytokines, steroid use, PN, chronic metabolic acidosis, and renal insufficiency may contribute to the development of metabolic osteopathy [74].
(II) Complications related with nutritional therapy	**Pathogenesis**
Thrombus-associated venous occlusion	Central venous catheter (CVC)-related thrombosis (CRT) is a severe complication of parenteral nutrition (HPN), which increases its associated morbidity (due to pulmonary embolism) and mortality rates of this population [75,76].
Catheter-associated central line bloodstream infections	Primary and intravascular catheter-associated bloodstream infections represent an important clinical entity in the intensive care unit (ICU) and has a poor effect on outcomes. Over-abundant levels of *Proteobacteria* have been found in the feces of patients with SBS presenting with Ca-CLBI [77,78,79,80].
IF-associated liver disease (IFALD)	IFALD is a possible complication in patients with IF who need intravenous support for survival due to severe intestinal dysfunction. An elevation of aminotransferases or cholestasis enzymes in this setting should raise clinical suspicion of this entity, which may progress from hepatic steatosis to cirrhosis. Some factors that increase the risk of this condition are shown in Figure 6. Liver cholestasis can be a life-threatening complication during HPN and may lead to a combined liver–intestinal transplantation (Figure 6) [17,79,81,82].
Re-feeding syndrome (RFS)	The switch from a catabolic state to an anabolic state in malnourished patients undergoing refeeding (orally, enterally, or parenteral) may be the cause of all these clinical manifestations, which, in some cases, can lead to death. RS include a complex and extensive list of changes, such as hypophosphatemia, hypomagnesemia, hypokalemia, hyponatremia, hypocalcemia, hyperglycemia, and vitamin deficiency (especially thiamine deficiency), all of which are accompanied by clinical signs and symptoms, reflecting organ dysfunction (cardiovascular, renal, respiratory, and neurological manifestations, among others). Figure 7 and Figure 8 summarize the relationship between the pathophysiology of RS and its clinical presentation [83,84,85,86,87,88,89,90].

**Figure 6:**
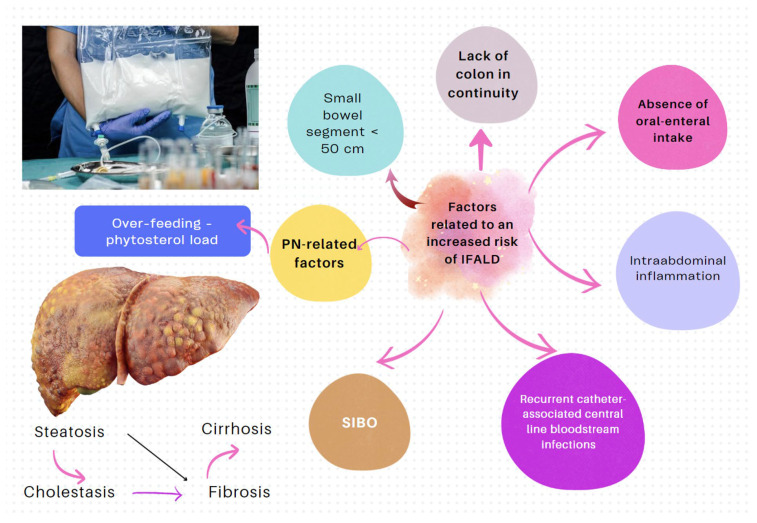
Factors influencing the development of IFALD [79,80,81,82].

**Figure 7:**
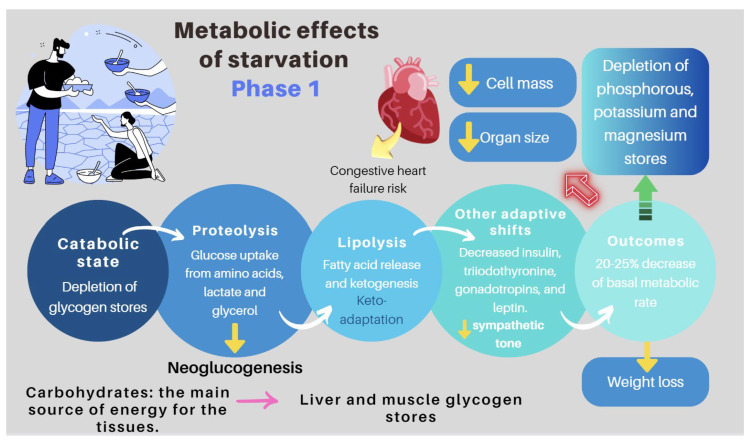
Pathogenesis of refeeding syndrome. Phase 1: the effects of starvation [83,86].

**Figure 8:**
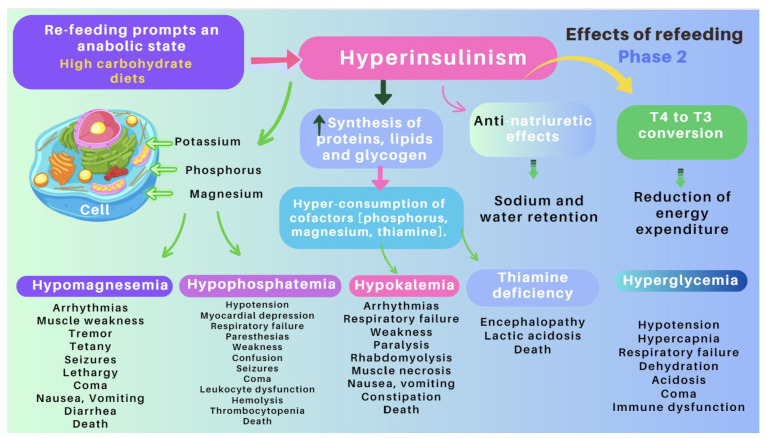
Pathogenesis of refeeding syndrome. Phase 2: Clinical and metabolic consequences of non-cautious re-feeding [83,86].

#### 6.1.2. D-lactic Acidosis

D-lactic acidosis is an unusual complication that mainly occurs in patients with malabsorption due to SBS or after a bariatric surgery. Colonic bacteria may degrade a surplus of fermentable carbohydrates to form D (−) lactate, which is absorbed but not easily metabolized and leads to severe encephalopathy of metabolic origin. D-lactic acidosis only occurs in patients with SBS and colon continuity and requires the following conditions: (1) colonic bacterial flora of a type that produces d-lactic acid; (2) ingestion of significant amounts of carbohydrates; (3) diminished colonic motility, allowing time for nutrients in the colon to undergo bacterial fermentation; and (4) impaired D-lactate metabolism [67,68,69]. Clinical manifestations of this condition include episodes of encephalopathy and metabolic acidosis. Its diagnosis should be considered in the presence of metabolic acidosis and neurological symptoms that cannot be attributed to other causes. Serum levels of more than 3 mmol/L of D-lactate are required to verify the diagnosis. Its treatments include the correction of metabolic acidosis by intravenous bicarbonate, restriction of carbohydrates or fasting, and antibiotics to eliminate intestinal bacteria that produce D-lactic acid [67,68,69].

#### 6.1.3. Refeeding Syndrome (RS)

RS is a rare, but severe, and potentially fatal complication related to the re-feeding of individuals who have fasted or consumed very few calories over a long period of time (83–87). Its clinical features comprise fluid-balance abnormalities, abnormal glucose metabolism, hypophosphatemia, hypomagnesemia, and hypokalemia. In addition, thiamine deficiencies can occur. The switch from a catabolic state to an anabolic state may be the cause of the clinical manifestations of RFS and justifies all the clinical features of this often severe and devastating clinical condition (Figure 7 and Figure 8) [88,89]. 

### 6.2. Basic Principles of Nutritional Management

**Figure 9:**
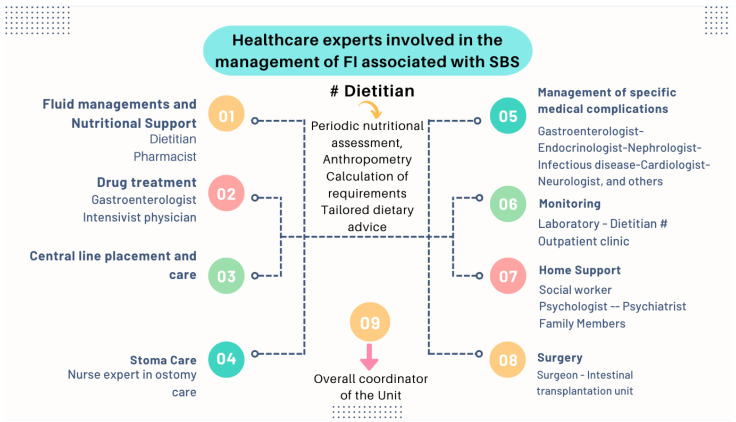
The management of patients with chronic IF requires an interdisciplinary approach through management by intestinal rehabilitation centers as the standard of care. This figure shows the team members involved in the overall care of these patients [93].

**Figure 10:**
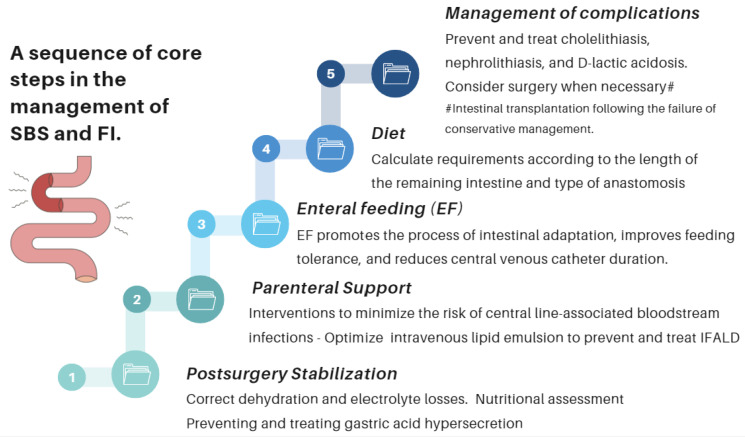
Essential strategic issues for the management of SBS and IF.

**Figure 11:**
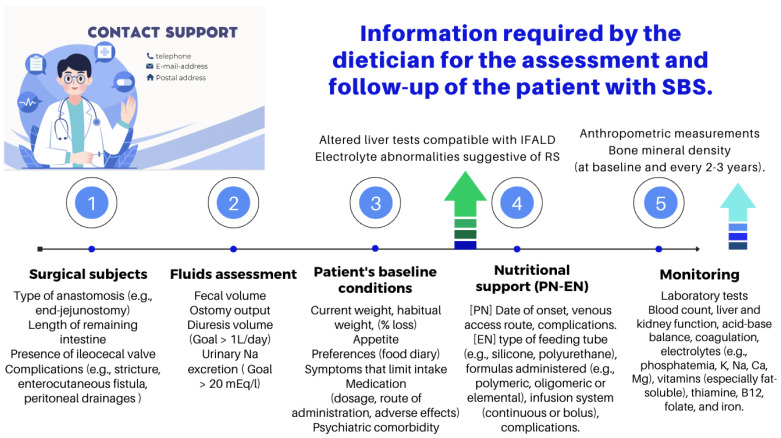
Functions of the dietitian in the nutritional assessment and follow-up of the patient with SBS [91,95]. PN: parenteral nutrition; EN: enteral nutrition; IFALD: IF-associated liver disease; and RS: re-feeding syndrome.

The treatment of patients with IF is complex and requires a comprehensive approach that frequently necessitates the long-term, if not life-long, use of PN. The appropriate management of this disorder requires an interdisciplinary approach to facilitate intestinal rehabilitation in combination with the treatment of the sequelae of IF [73]. Recently, guidelines and recommendations have been developed by the American Gastroenterological Association (AGA) [55], ASPEN [85,90], and ESPEN [91]. Although an extensive review is beyond the scope of this manuscript, it is essential to highlight some basic concepts:

- Nutritional interventions to treat SBS include enteral and PN, intestinal rehabilitation techniques to increase the absorptive ability of the residual bowel, and surgical reconstruction designed to enhance the surface area for absorption [92,93,94]. Therefore, managing these patients requires a comprehensive and interdisciplinary approach in centers with proven experience in treating such challenging scenarios [93,94,95,96,97]. This issue is essential, as has been demonstrated by Geransar P et al., who reported a low level of awareness of chronic IF among non-specialist healthcare professionals [95]. Figure 9 highlights the components of a multidisciplinary team caring for these patients in a highly specialized center [93,96], and Figure 10 outlines the fundamental steps to be followed in the assessment and treatment of these patients [97].

- The role of an experienced dietitian is also fundamental in the initial assessment and monitoring of the nutritional status of patients with SBS. Concerning this point, the dietitian should obtain information regarding multiple details related to the type of surgery performed, the patient’s baseline post-operative conditions (renal function, water–electrolyte, and acid–base balance), their degree of malnutrition, and the type of nutritional support received (enteral or parenteral), including access routes, as well as their associated complications (Figure 10 and Figure 11).

- Almost all patients with SBS need PN during the early period after a resection. PN should be initiated and adjusted to meet the patient’s fluid, electrolyte, energy, protein, and micronutrient needs. The literature provides excellent reviews of PN performance in this setting [98,99,100,101]. Although few patients with severe SBS can discontinue PN before hospital discharge, more than 50% of adults will be able to wean completely from PN within five years of their diagnosis. PN volume can be decreased when the patient begins to tolerate oral nutrition. This is possible if the volume or flow of feces from the rectum or through the ostomy is adequately reduced and the patient begins to gain weight. Enteral nutrition (EN) provides clear benefits, prevents villous atrophy, helps preserve the intestinal epithelial barrier, enhances the local immunity needed to avoid SIBO, and promotes the mechanisms of bowel adaptation, enhancing weaning from parenteral nutrition. The introduction of EN should always be prudent and judicious [100,101]. One approach is to start EN by providing 5% of the total calories and increasing this ratio every 3–7 days and assess tolerance. Patients who require long-term PN cannot be kept in hospital indefinitely, and thus transition to home PN when they are clinically stable. To maximize patient mobility and convenience at home, PN infusion time is minimized, and the solution is infused overnight. PN infusion time can typically be reduced (cycled) to 10–15 h, depending on patient tolerance [96,97]. Notably, the SBS patient receiving home PN is still at risk of micronutrient deficiencies, as well as liver and bone complications, and requires regular monitoring and supplementation with PN (Table 6).
- Many patients with less severe forms of SBS may be fed orally early. The dietary and nutritional management of these patients necessitate to understand the physiology and to consider the individual anatomy and adaptation phase. During the hypersecretory phase, fluid losses are usually the largest. Dehydration and saline depletion can occur during any phase, especially in patients without a colon, and particularly in case of an end jejunostomy [98].

### 6.3. Recommendations of Scientific Societies

ASPEN has issued specific dietary advice and recommendations for the above phenotypes (ASPEN, www.nutritioncare.org). We outline some key messages about these recommendations:

- **Type 1: end jejunostomy.** This is the most unfavorable phenotype, as malabsorption is more severe, and it presents with a high ostomy output. Patients without a colon and <100 cm of the jejunum have a higher risk of requiring long-term PN. Indeed, dehydration, hydroelectolytic abnormalities, acidosis, and renal failure are more likely in these patients [57]. Sodium absorption in the jejunum is dependent on water fluxes and is coupled to the absorption of glucose. For this reason, hydration with hypotonic solutions (e.g., water, tea, or coffee) should be discouraged, as they only exacerbate fluid losses through the stoma. Hypertonic drinks (e.g., fruit juices) should also not be recommended as they cause osmotic diarrhea. Some measures that may be useful for these patients are lowering the intake of sugars, decreasing the size of intakes, and take the oral rehydration solutions (ORSs) whose composition is best suited to promote the entry of sodium and water into the enterocytes [102,103]. Regarding calorie and macronutrient requirements, the recommendations are as follow (Table 6):

- **Type 2: jejunocolic.** It retains a portion of the jejunum anastomosed to a portion of the colon. In these patients, the clinical picture is dominated by diarrhea due to severe malabsorption, vitamin–mineral deficiencies, and subsequent malnutrition. Patients with jejunocolic anastomosis and <50 cm of the jejunum also have a higher risk of requiring long-term PN. The nutritional recommendations for those who recover intestinal autonomy are as follows (Table 7):

- **Type 3: jejunoileocolic.** These patients retain their entire colon and ileocecal valve along with a portion of their terminal ileum and jejunum. This is indeed the most advantageous phenotype, and these patients often do not require additional nutritional support because the ileum has a greater ability to adapt. This subgroup does not usually develop malnutrition, electrolyte disorders, or dehydration [57].

### 6.4. Common Recommendations for All Three Phenotypes

The intestinal mucosa usually absorbs lactose unless it is affected by lesions (e.g., Crohn’s disease), leading to villous atrophy and a secondary lactase deficiency. Therefore, lactose restriction is not justified, as it is a natural protein, calcium, and vitamin D source. With respect to the amount of fiber, 10–15 g/day is recommended (depending on individual tolerance). Patients with a fecal fluid excretion level >3 L/24 h may require 5–10 g of soluble fiber per day.

### 6.5. Vitamin and Mineral Replacement

As water-soluble vitamins are absorbed in the proximal small bowel, deficiencies in SBS patients are uncommon, except in very extensive resections. Nevertheless, fat-soluble vitamin deficits are relatively frequent, and large doses may be required to maintain normal serum levels [50,57]. Consequently, in all patients with SBS, serum vitamin and trace element concentrations should be measured at baseline and monitored on a regular basis (Table 8) [50,57,104,105,106,107,108,109,110].

### 6.6. Pharmacological Treatment

Regarding the most prescribed pharmacological treatments for SBS, anti-secretory medications (e.g., proton-pump inhibitors or histamine H2 receptor antagonists) can reduce gastric acid secretion after a massive bowel resection in order to decrease fluid and electrolyte losses. They are generally used for periods of up to 6 months [111,112]. Antimotility medications, such as loperamide (2–8 mg, 30–60 min before meals and bedtime) and occasionally codeine phosphate (30–60 mg), are usually used to enlarge the intestinal transit time and therefore prevent the loss of large volumes of fecal fluid. Treatment should be initiated with a single first-line medication at the low end of its dosing range. Dosage and/or dosing frequency can then be slowly escalated (every 3–5 days) to achieve maximal effects while minimizing adverse events. Codeine phosphate is a potent anti-diarrheal agent, but it should be used cautiously due to its CNS-acting effects and addictive properties [57,113]. 

Antimicrobial agents, such as rifaximin (550 mg, three times a day), metronidazole (250–500 mg, three times a day), or doxycycline (100 mg, twice a day), administered periodically may be helpful in neutralizing the effects of SIBO, a condition associated with various gastrointestinal symptoms, as well as nutrient deficiencies, and weight loss [114,115]. It has recently been shown that the bioavailability of oral antibiotics was higher than expected in patients who have suffered a loss of their anatomical surface area for absorption [116].

Other emerging therapies may include probiotics, diet manipulation, and prokinetic agents. Bile acid sequestrants (e.g., cholestyramine or colesevelam) can be used in patients with bile acid diarrhea due to limited ileal disease or resection. However, they can worsen steatorrhea in SBS, and should be discouraged in patients with ileal resections >100 cm [8,10,113,117,118,119,120]. The role of the pancreatic enzyme replacement treatment in this scenario has not been sufficiently elucidated [112,121]. Ursodeoxycholic acid (UDCA) treatment decreases the hepatic synthesis of triglycerides and cholesterol. Recent studies have reported that UDCA (20 mg/kg/day) could prevent the onset of IFALD by decreasing hepatic lipogenesis [122].

Somatostatin and octreotide reduce salivary, gastric, and pancreaticobiliary secretions, slow small bowel transit, and may delay gastric emptying; for these reasons, they reduce the intestinal output from a jejunostomy in both net ‘secretors’ and ‘absorbers’. Studies involving adults have shown octreotide to reduce ileostomy diarrhea and large- volume jejunostomy outputs (2 L/d) [123,124]. Some problems associated with the long-term use of octreotide are: (1) A lower number of amino acids for splanchnic protein synthesis. This may interfere with the physiological process of adaptation to an intestinal resection [123,124]; (2) a reduction in pancreaticobiliary secretion, worsening fat absorption [125,126,127]; however, it is usually unchanged [128,129,130]; (3) a higher risk of cholelithiasis [123,124,131,132,133]. The effectiveness of other long-acting somatostatin analogues, such as lanreotide, has not been sufficiently tested in this setting [134].

Teduglutide reduces gastric emptying and secretion and may promote the growth of the mucosa [135,136,137,138,139,140,141,142]. Studies over the last five years have shown that teduglutide, in addition to reducing the volume and calories administered through parenteral support, also reduces infusion days, sleep disturbances, stable oral intake alternatives, and improved stool characteristics [143,144,145,146,147]. Growth hormone (GH) activates the proliferation of intestinal stem cells (ISCs), enhances the formation of crypt organoids, and drives the differentiation of ISCs into Paneth cells and enterocytes. Glutamine (GLN), on the other hand, also enhances the proliferation of ISCs [148]. It has been hypothesized that the administration of growth factors and/or nutrients could enhance further compensation of the remnant intestine and thereby improve absorption. Specifically, animal studies have shown that there is enhanced cellularity with the administration of GH or GLN or a fiber-containing diet. A retrospective evaluation of 17 studies carried out in humans with SBS showed an improvement in protein absorption by 39% and a 33% decrease in stool output with the GH + GLN + DIET (high-carbohydrate, low-fat diet). In the long-term study, 40% of the group remained off PN, and an additional 40% had reduced their PN requirements [149]. More recent studies have suggested that the benefits of administering recombinant human GH alone, or together with GLN with or without a low-fat diet containing a high level of carbohydrates (fiber), are, if any, marginal, and there are concerns about their potential long-term risks [150].

### 6.7. Management of Other Specific Conditions

**Figure 12:**
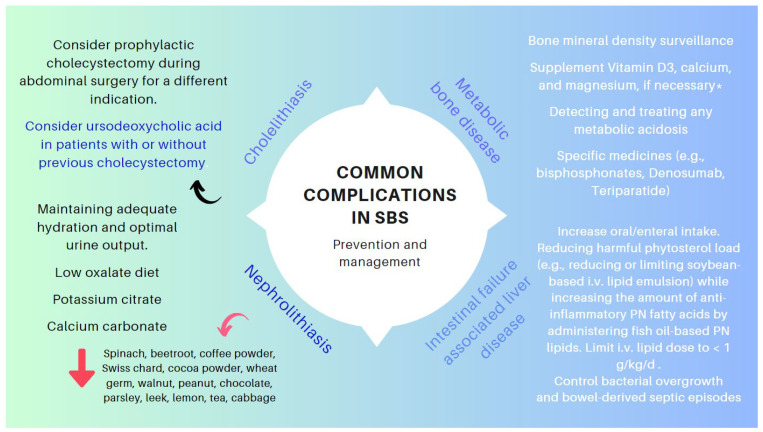
Key points in the management of specific complications associated with SBS. * SBS leads to gastrointestinal losses of magnesium. Hypomagnesaemia (mainly if Mg < 1.5 mg/dL) often leads to genuine hypocalcemia by blocking parathormone secretion and promoting parathormone resistance. Hence, the importance of correcting hypomagnesemia so as not to aggravate metabolic osteopat.

The clinical management of the patients with IF and SBS includes preventing and treating a range of complications related to the pathophysiology of the small and large intestines (e.g., nephrolithiasis [72,73,74], cholelithiasis [70,71], and metabolic bone diseases [74]) or to the artificial nutritional support systems (IAFLD and catheter-related bloodstream infections). Figure 12 outlines the critical points in the management of these conditions [82,151,152].

### 6.8. Surgical Management

Conservative management remains the first-line approach for patients with SBS. Nevertheless, various nontransplantation surgical procedures have a role in improving intestinal function in SBS and have shown their effectiveness in properly selected patients. These surgeries focus on slowing down intestinal transit, to increase contact time between nutrients and the mucosa, to correct remnant bowel dilation and stasis, to improve intestinal motility, and to increase mucosal surface area. The type of procedure is selected depending on the age, length, and functionality of the remnant bowel, the existence of intestinal dilation, the presence of SIBO, and the presence or absence of PN-related complications. The most common procedures include:

(1) Preserving the existing intestine: It is common that after the initial resection, some patients need to be re-operated for various reasons (e.g., stenosis and perforations). In these scenarios, avoiding a resection and preserving the existing length of the intestinal remnant (e.g., serosal patching for certain strictures and chronic perforations) are essential. When carrying out a resection becomes unavoidable, an end-to-end anastomosis is preferred to prevent blind loops and, thus, optimize the functionality of the hindgut [58].
(2) Restoration of intestinal continuity, elimination of a stoma with the aim of improving the patient’s quality of life and avoiding some of the complications associated with central venous catheters [57].
(3) Tapering surgery when the remaining small bowel remains excessively dilated [153]. Intestinal tapering may be necessary in this context as a dilated intestine increases the risk of mucosal injury, bloodstream infections, and liver disease in patients with SBS [154]. Several techniques have been described to taper the dilated small bowel, including longitudinal intestinal lengthening and tapering, serial transverse enteroplasty, and spiral intestinal lengthening and tailoring [153,155].
(4) Correction of stenoses, if possible, with stricturoplasties and with remodeling or intestinal plication if needed [156].
(5) Serosal patching for chronic fistulae to prevent avoidable intestinal excisions [157].
(6) Autologous gastrointestinal reconstruction operation: The aim of this procedure is to either enhance the mucosal surface area for absorption (e.g., lengthening procedures) or to slow intestinal transit to facilitate the assimilation of the nutrients or counterbalance stasis that cause gastrointestinal symptoms due to SIBO (e.g., reversing the segments of the intestine) [158,159], creating intestinal valves, or interposing a colonic segment in the mall intestinal remnant in either an isoperistaltic or antiperistaltic fashion [7,160,161,162]. These procedures should only be used in carefully selected patients and in centers with proven experience [163,164,165].

New surgical methods are currently under development, including spring-mediated distraction enterogenesis (implantation of self-expanding springs through an endoscopic intervention) [166] and a set of techniques focused on regenerative medicine, opening up the possibility of repairing and replacing intestinal tissue on demand. The latter include a variety of tissue-engineered small intestines (TESIs). TESI approaches range from small intestinal submucosa grafts to intestinal tissue repurposing with either stem cells or organoid units, the latter of which appears extraordinarily promising [163,167,168,169,170]. 

### 6.9. Intestinal Transplantation (ITx)

ITx can be lifesaving and can improve the quality of life for patients with irreversible IF. The long-term results of ITx are not as good as other types of visceral transplants (e.g., liver, heart, or kidney). The intestine is a complex organ to transplant due to its immunogenicity, large population of donor immune cells present within the graft, and its nonsterile contents. Rejection causes barrier failure and bacterial translocation, so sepsis may occur when increased immunosuppression is required [171]. Thus, unlike renal failure, ITx cannot yet be recommended as an alternative therapy for patients stably maintained on intravenous nutrition. Unfortunately, the outcomes following ITx are not optimal, with 10−20% of patients continuing to be dependent on, at least, partial PN and survival at ten years post-transplant of only 40% with a lower graft survival rate [172,173,174].

Scientific societies have formulated guidelines and recommendations focused on the need to balance the advantages and disadvantages of ITx [91,171,173,175,176,177,178]. Following this postulate, ESPEN published, in 2016 [172] and 2021 [91], specific recommendations regarding the indications for ITx in patients with SBS, which have ultimately been updated in 2023 [178]. Patients with SBS–IF with high morbidity or a low acceptance of PN should be considered for early listing for ITx on a case-by-case basis [172,173,174,175,176,177,178]. So, threatening complications warranting the consideration of intestinal Tx include IAFLD, recurrent sepsis, and threatened loss of central venous access. Table 9 shows the revised criteria for placement on a waitlist for ITx, presuming that a multidisciplinary team will have assessed patients, explored rehabilitation options, and a state of permanent or life-limiting intestinal failure exists [17,91,176,177,178,179,180].

## 7. Anorectal Surgery

Benign conditions affecting the anus and rectum, such as abscesses and fistulas, fissures, hemorrhoids, or condylomas, are among the most common digestive diseases. These conditions can cause various symptoms, such as pain, itching, burning, bleeding, suppuration, and/or fever, and swelling in the most severe cases. However, patients may only request their evaluation when their lifestyle is significantly affected. Anal surgery is usually performed under sedation and local anesthesia or spinal anesthesia and leads to a short hospital stay, either ambulatory or 24-h stay [181,182]. Therefore, the priorities for post-operative assessments should be to correct analgesia and defecatory habits to prevent the appearance of the aforementioned symptoms. 

Both the appearance of diarrhea and constipation can worsen the healing process and quality of life after anorectal surgery. Consequently, irritating foods, such as acidic or spicy foods and caffeine, should be avoided. Prioritizing hydration and high-fiber foods (such as raw fruits, vegetables, and whole-grain bread) will result in softer, bulkier stools, helping to prevent constipation and straining [183,184]. For this reason, bulk-forming agents, and lubricant laxatives (e.g., liquid paraffin or glycerin) are not only recommended but are also associated with earlier and less painful defecation following anal surgeries [185,186,187]. It is important to highlight that incontinence symptoms such as fecal leakage or soiling appear and/or worsen less frequently with soluble fiber than in osmotic laxative regimes [188]. 

Other therapeutic strategies, such as medical bowel confinement or parenteral nutrition, have not been shown to offer benefits in clinical and patient-reported outcomes after various anorectal operations [187,188,189].

## 8. Conclusions and Highlights

### 8.1. Ileocecectomy

The first and most important therapeutic measure for chronic bile acid diarrhea has been the administration of bile acid sequestrants (e.g., cholestyramine/cholesevelam). After starting this therapy, patients report less frequent and more solid stools, leading to the disappearance of incontinence and fecal urgency. However, constipation, abdominal distension, cramps, or nausea may appear, requiring dose adjustment until finding the best that can be tolerated. As bile acid sequestrants can bind to other compounds, chronic treatment with cholestyramine may lead to deficiencies in fat-soluble vitamins (e.g., vitamins A, D, E, and K), so periodic measurement and dietetic support is recommended. In ileal resections >100 cm, cholestyramine may exacerbate steatorrhea and worsen diarrhea. 

### 8.2. Low Anterior Resection and Abdominoperineal Resection

Patients with diversion colitis may be treated with short-chain fatty acid enemas, topical 5-aminosalicylic acid agents, topical glucocorticoids, and proctectomy or sigmoid colectomy with proctectomy for refractory symptoms. Patients with LARS may benefit from dietary intervention. Foods high in soluble fiber should increase the consistency of solid stools, thereby improving incontinence symptoms due to diarrhea. Eating soluble fiber (bulking agents) attracts water and should be encouraged to better stool consistency. In contrast, insoluble fiber may worsen symptoms by increasing the number of spontaneous defecations, the bulk of the stool, and causing bloating. Dietetic input is essential for fiber balancing and for ensuring that the diet remains nutritionally adequate. 

### 8.3. Proctocolectomy

Patients arrive in the operating room under deleterious nutritional conditions due to protein-losing colopathy, anemia, and metabolic stress associated with SRIS. This complex scenario requires the appropriate peri-operative nutritional support to optimize the outcomes. Following a total colectomy, it is expected to have a high intestinal fluid loss and metabolic derangement. In the acute stage, intravenous fluid replacement with normal saline (0.9 percent) and supplemental potassium and magnesium are crucial. Hypertonic fluids (e.g., fruit juices) should be avoided, as they contribute to osmotic diarrhea. The colon absorbs up to 15 percent of the daily energy needs in healthy adults. Therefore, the loss of the colon not only involves a loss of fluids and electrolytes but also energy. In the absence of the colon, a diet rich in simple carbohydrates can be detrimental because concentrated carbohydrates have a high level of osmolarity, which can lead to diarrhea.

### 8.4. Stoma Formation

#### 8.4.1. Ileostomy

Dehydration is the most common cause of hospital readmission after ileostomy surgery. The first-line management of patients with elevated ileostomy outputs (defined as >1.5 L/day) should include gel-forming fiber supplementation (e.g., psyllium husk), which can slow the transit time by absorbing water and forming a gel-like consistency. Another issue with the dietary implications for these patients is gas production from carbohydrate fermentation. The dietitian can provide valuable support by informing these patients about some foods containing raffinose, a trisaccharide composed of glucose, fructose, and galactose that is fermented by bacteria in the intestinal lumen and increases gas production (Table 2 and Figure 3). Notes should be made on the common nutritional deficiencies that arise in people living with an ileostomy, with vitamin B12 deficiencies affecting 31%. If a B12 deficiency does occur, this needs to be corrected with IM injections, as the site for absorption in the terminal ileum will not be able to function.

#### 8.4.2. Colostomy

Patients with a colostomy should be encouraged to ingest sufficient amounts of fiber (20 g/day to 35 g/day) and fluids (at least 1.5 L to 2 L/day) to prevent constipation and should also be counseled regarding gas-producing foods and lag times. A balanced diet and properly conducted nutritional education are crucial in avoiding peristomal complications and several nutritional deficiencies.

### 8.5. IBD Surgery

The impact of IBD on nutritional status can be severe, ranging from generalized weight loss and growth failure to deficiencies in specific vitamins and trace elements, especially vitamin D3, B12, folate, calcium, iron, zinc, selenium, copper, and magnesium, and increased post-operative morbidity of those patients undergoing a surgery due to local or systemic complications of their IBD. The ESPEN recently renewed the clinical practice guidelines on the nutritional management of IBD by formulating up to 71 recommendations based on the best scientific evidence [50]. It addresses a wide range of clinical scenarios, including considerations of peri-operative nutritional risk, indications for nutritional support, and nutritional strategies for specific contexts (e.g., high-output ostomy, stricture, or proctocolectomy with extensive fluid and electrolyte losses). Again, the input of the dietitian is essential to improve the outcomes and potentially the costs of this approach.

### 8.6. Short Bowel Syndrome

The clinical presentation of SBS comprises a range of intestinal and extraintestinal manifestations, depending on the impairment of absorption and/or stimulation of water and electrolyte secretion, metabolic disturbances inherent to the nutritional support (e.g., IFALD, D-lactic acidosis, and RS), or the insertion of central catheters. The magnitude and nature of these complications depend on several factors, particularly the type of anastomosis performed: jejunum–colon, jejunum–ileum, and end jejunostomy. 

Managing these patients requires a comprehensive and interdisciplinary approach in centers with proven experience in treating such challenging scenarios (Figure 9). The guidelines and recommendations on this matter have been developed by the AGA [55], ASPEN [85,90], and ESPEN [91]. Again, the role of an experienced dietitian is essential to calculate the requirements according to the extent of the remaining intestine and the type of reconstruction (Figure 10), as well as providing tailored dietary advice to improve clinical outcomes and quality of life.

### 8.7. Anorectal Surgery

Irritating foods, such as acidic or spicy foods and caffeine, should be avoided after an anorectal surgery due to benign conditions affecting the anus and rectum. Prioritizing hydration and high-fiber foods (such as raw fruits, vegetables, and whole-grain bread) will result in softer, bulkier stools, helping to prevent constipation and straining.

## Figures and Tables

**Table 1:** Dietary intervention in the patient with LARS.

Dietary Advice	Background and Potential Benefits
Avoiding foods that contribute to decreased stool consistency (e.g., caffeine, plums or plum juice (both contain sorbitol), alcohol, and excessive fat).	Avoiding these foods helps to improve stool consistency (e.g., Bristol 6–7 to Bristol 5) and improves continence.
Intake of high-fiber foods.	High-fiber foods should lead to an increase in solid stool consistency, thus improving symptoms or incontinence due to diarrhea.
Avoiding an over-ingestion of insoluble fiber (e.g., wheat bran, some fruits (e.g., kiwi, grapes, raspberries, strawberries, rhubarb, pineapple, raisins, and blueberries), vegetables (e.g., green beans, corn, eggplant, broccoli, kale, spinach, and legumes like chickpeas or lentils), and whole grains).	Insoluble fibers add bulk to the stool and appears to help food pass more quickly through the stomach and intestines. However, they could lead to a deterioration of symptoms due to an increased number of spontaneous defecations as well as bloating #.
Preferably eat soluble fiber (bulking agents) (e.g., oat bran, nuts, barley, peas, seeds, beans, lentils, some fruits (e.g., guavas, apples, nectarines, pears, avocados, and apricots), and vegetables (e.g., brussels sprouts, broccoli, sweet potatoes, carrots, and turnips), as well as the psyllium, a common fiber supplement.	Soluble fibers attract water and turn into a gel during digestion. This slows digestion. In addition, soluble fibers should be recommended to achieve better stool consistency #.

LARS: low anterior resection syndrome. # High-residue foods in conditions like LARS lead to bulkier stools that are more challenging for those with rectal dysfunction, while soluble fibers lead to softer and more uniform stools that are easier for a compromised rectum to pass.

**Table 2:** List of foods that contribute to symptoms by increasing gas production.

Some Foods that Cause Abdominal Bloating due to Their Increased Production of Hydrogen, Carbon Dioxide, and Methane Gas.
Due to their high raffinose content:
*beans*, *asparagus*, *soybeans*, *chickpeas*, *peanuts*, *peas*, *beans cabbage*, *cauliflower*, *brussels sprouts*, and *broccoli.* Their consumption can cause flatulence because the tract does not synthesize α-galactosidase, the enzyme responsible for hydrolyzing these oligosaccharides.
Due to their high starch content: *potatoes*, *corn*, *noodles*, and *wheat*, but *not rice*.
Due to their high soluble fiber content: *carrots*, *celery*, *onion*, *oat bran*, *peas*, and *most fruit*, including *apples*, *pears*, *peaches*, *plums*, *figs*, *cherries*, and *dates*.
**Some foods that decrease gas production, contributing to the attenuation of symptoms:** *Pineapples* (for their bromelain content (a proteolytic enzyme)), *papayas* (contains papain), *kiwis* (actidine), and even *figs*, rich in ficin.

**Table 3:** Reasons that may explain malnutrition in patients with IBD who may require surgery.

Mechanisms Involved in the Malnutrition of IBD Patients.
Limited intake caused by outbreaks of inflammatory activity (coexistence of limiting symptoms), and hypoorexia associated with the release of interleukins (e.g., IL-1 and IL-6) and tumor necrosis factor alpha.
Malabsorption caused by enteropathy (e.g., focal and segmental villous atrophy).
Maldigestion (e.g., steatorrhea due to malabsorption of bile salts after ileum resections >100 cm or secondary disaccharidase deficiency in the presence of severe enteropathy).
Bowel obstruction (nausea, vomiting, and inability to ingest food).
Protein-losing enteropathy due to malabsorption and mucosal ulceration (exudation of blood, mucus, and proteins) or high-output enterocutaneous fistulas.
The anatomical absorption surface is reduced after extensive small bowel resections, especially if the length of the removed ileum is beyond the critical point of 100 cm.
Increased basal energy expenditure due to transient states of catabolism (e.g., suppurative complications, such as fistulas and abscesses, leading to SIRS), severe outbreaks of ulcerative colitis with systemic toxicity, or major surgery.
Small intestine bacterial overgrowth (bacteria cause focal and segmental villous atrophy, deconjugation of bile salts, and excessive consumption of vitamin B12).
Glucocorticoids (interference with growth hormones, bone formation, nitrogen retention, and collagen synthesis).
Adverse effects of other drugs: metronidazole: decreased palatability; methotrexate/sulfasalazine: <bioavailability of folate).

IBD: inflammatory bowel disease; SRIS: systemic inflammatory response syndrome.

**Table 4:** Multifactorial causes of diarrhea in SBS.

Mechanism Explaining Diarrhea in SBS	Comment
Loss of the anatomical absorption surface.	The pattern of nutrient absorption native to the parts of the gastrointestinal tract is shown in Figure 1.
Increase in the volume of hydrochloride secretion due to a failure of gastric secretory inhibitory mechanisms via the defective release of enterogastrones (e.g., VIP, GIP, neurotensine, peptide YY, and GLP-1).	Adds a great volume of secretions to the upper digestive tract. An excessively acidic pH denatures pancreatic enzymes and interferes with the action of bile salts, causing maldigestion.
Accelerated gastric and intestinal transit by disrupting the feedback mechanism depends on hormone release from the ileum (see above).	Maldigestion due to inadequate mixing of pancreatic enzymes and biliary salts with the macronutrients.
Decreased bile salt pool in the intestinal lumen caused by the resection of the ileum and interruption of enterohepatic circulation.	Choleretic diarrhea if resection <100 cm ^#^. Steatorrhea and malabsorption of liposoluble vitamins (e.g., vitamins A, D, K, and E) if resection >100 cm ǂ.
Small intestinal bacterial overgrowth (SIBO).	SIBO injures the enterocytes, causing focal and segmental villous atrophy and secondary lactase deficiency, resulting in osmotic and secretory diarrhea.
Presence of inflammation in the remaining intestine (e.g., IBD or radiation injury).	Inflammatory diarrhea with exudation of blood, mucus, and pus, as well as protein-losing enteropathy.
*Clostridium difficile* infection.	Substantial risk in patients who receive antibiotics due to central catheter infections and/or remain bedridden postoperatively.
Inappropriate intake of hypotonic solutions (e.g., water, tea, or caffeine) or hyperosmolar drinks (e.g., fruit juices or sports drinks) for thirst relief, especially in patients with an end jejunostomy.	Jejunal mucosa is “leaky” and rapid sodium. fluxes occur across it. If water or any solution with a sodium concentration of less than 90 mmol/L is drunk, there is a net efflux of sodium from the plasma into the bowel lumen, exacerbating diarrhea.
Diarrhea-causing drugs or oral nutritional supplements with high osmolarity.	E.g., omeprazole, non-steroidal anti-inflammatory drugs, psychotropic drugs, colchicine, or resin cholestyramine in patients with ileal resections >100 cm.

^#^: diarrhea improved with cholestyramine; ǂ: diarrhea often worsens with cholestyramine.

**Table 6:** Nutritional recommendations for the “end jejunostomy” (ASPEN).

Requirement		Comment
Energy	35–45 kcal/kg/day. In some cases, increasing the energy intake up to 60 kcal/kg/day may be necessary.	Patients with SBS develop compensatory hyperphagia, and it is advisable to take 5–6 meals spaced out during the day.
Carbohydrates	20–40% of the daily energy target.	In the absence of a colon, it is not possible to rescue energy inherent in the production of short-chain fatty acids from the bacterial fermentation of sugars.
Protein	1.5–2.0 g/kg/day or 20–30% of the daily energy target.	It is preferable to choose lean proteins of high biological value.
Fat	40–60% of the daily energy target.	Choose essential fatty acids as the main component of fat intake. Consider MCTs in cases of malabsorption.
Fluids	Reduce oral hypotonic fluids to 500 mL/day #. Separating solids and liquids (i.e., do not drink anything for half an hour before or after a meal).	Add sodium chloride to any liquid feeds to make the sodium concentration near 100 mmol/L while keeping osmolality near 300 mOsmol/kg *.
Oxalate	The restriction is not necessary.	Calcium oxalate stones only occur in patients with a preserved colon.

* Administer glucose/saline solution in small sips (sodium concentration of at least 90 mmol/L). Most stomal/fistula leakages (except from the colon) have a sodium concentration of least 90 mmol/L. In cases of severe dehydration, administration of intravenous saline while the patient does not take anything by mouth for 24–48 h will stop thirst and therefore the desire to drink. # Osmolarity of drinks: hypertonic > 300 mOsmol kg^−1^; isotonic: 275–300 mOsmol kg^−1^; and hypotonic: <275 mOsmol kg^−1^.

**Table 7:** Nutritional recommendations for the jejunocolic anastomosis (ASPEN).

Requirement		Comment
Energy	35–45 kcal/kg/day. In some cases, increasing the energy intake up to 60 kcal/kg/day may be necessary.	Patients with SBS develop compensatory hyperphagia, and it is advisable to take 5–6 meals spaced out during the day.
Carbohydrates	50–60% of the daily energy target.	The colon provides energy (up to 1000 kcal/day) in SBS by releasing the SCFAs resulting from the fermentation of carbohydrates. In addition, it provides nutrition to the colonocytes.
Protein	1.5–2.0 g/kg/day or 20–30% of the daily energy target.	It is preferable to choose lean proteins of high biological value.
Fluids	Isotonic/hypotonic #.	In SBS, the colon plays a vital role in fluid and electrolyte reabsorption, given the additional fluid that enters the colon with a capacity to absorb up to 6 L daily.
Fat	20–30% of daily energy target.	In jejunum–colon patients, unabsorbed long-chain fatty acids in the colon are likely to reduce the transit time and reduce their water and sodium absorption, making their diarrhea worsen. Consider MCTs only in the case of severe malabsorption. MCT does not contain essential fatty acids.
Oxalate	The diet should be low in oxalate.	Nephrolithiasis only occurs in patients with a preserved large bowel.

# Osmolarity of drinks: hypertonic > 300 mOsmol kg^−1^; isotonic: 275–300 mOsmol kg^−1^; and hypotonic: <275 mOsmol kg^−1^.

**Table 8:** Long-term vitamin and mineral supplementation in SBS [50,57,105,108,109].

Type of Micronutrient and Average Nutritional Intake Ranges	Clinical Signs Reflecting a Deficiency	Measurement	Typical Supplementation in SBS *
***Water-soluble vitamins***			***Doses (all values per day)***
Vitamin B1—thiamine (DRI: 1.1–1.2 mg/day)	Mental changes (apathy, decrease in short-term memory, confusion, and irritability), cognitive deficits, congestive heart failure, and metabolic lactic acidosis	Whole-blood ThDP or RBCs	Oral: 1–2 capsules daily (multivitamin: B1, B2, B3 B5, B6, and B7)
			HPN and long-term PN: 2.5 mg/day
Vitamin B2—riboflavin (DRI: 1.3 mg/day—males. 1.1 mg/day—females)	Seborrheic dermatitis of the face, trunk, and scrotum, oral buccal lesions, ocular manifestations, marrow aplasia, and normochromic, normocytic anemia	Glutathione reductase activity in red blood cells	Oral: 1–2 capsules daily (multivitamin: B1, B2, B3 B5, B6, and B7)
			HPN and long-term PN: 3.6 mg/day
Vitamin B3—niacin (DRI: 16 mg/day—adolescents and adult males > 14 years. 14 mg/day—females > 14 years)	Dementia, dermatitis, and diarrhea	Blood or tissue NAD	Oral: 1–2 capsules daily (multivitamin: B1, B2, B3 B5, B6, and B7)
			HPN and long-term PN: 40 mg/day
Vitamin B5—pantothenic acid (DRI: 5 mg/day for 14 to over 70 years)	Fall in the diastolic and lability of systolic blood pressure, with postural hypotension, vertigo, and tachycardia. Gastrointestinal and neurological symptoms	Blood pantothenic acid	Oral: 1–2 capsules daily (multivitamin: B1, B2, B3 B5, B6, and B7)
			HPN and long-term PN: 10 mg/day
Vitamin B6—pyridoxine (DRI: 1.3–1.7 mg/day for 14 to over 70 years)	Microcytic anemia, seborrheic dermatitis with cheilosis and glossitis, angular stomatitis, epileptiform convulsions, confusion, and/or depression	PLP levels. Red cell PLP in serious patients or in the presence of inflammation	Oral: 1–2 capsules daily (multivitamin: B1, B2, B3 B5, B6, and B7)
			HPN and long-term PN: 4 mg/day
Vitamin B7—biotin (DRI: 40 μg/day)	Ataxia, dermatitis, and alopecia	Direct measure of urine and blood biotin that must be completed with the determination of biotin activity	Oral: 1–2 capsules daily (multivitamin: B1, B2, B3 B5, B6, and B7)
			HPN and long-term PN: 60 μg/day
Vitamin B9—folic acid (DRI: 330 μg/day DFE)	Glossitis, megaloblastic anemia, pancytopenia, oral ulcers, angular stomatitis, and neuropsychiatric manifestations	Folate level in the plasma or serum—short-term status. In RBCs—long-term status	Oral: 1 mg daily
			HPN and long-term PN: 400 μg/day
Vitamin B12—cobalamin (DRI: 2.4 μg/day)	Hematological, neurological, neuropsychiatric, and cognitive symptoms	Combination of at least two bio- markers (e.g., holo-TC and MMA), with serum cobalamin as a replacement for holo-TC when the measurement of this latter is unavailable	Oral: 1–2 capsules daily (multivitamin: B1, B2, B3 B5, B6, and B7)
			HPN and long-term PN: 5 μg/day
Vitamin C—ascorbic acid (DRI: 90–100 mg/day)	Lassitude; shortness of breath; anemia; poor wound healing; myalgia and bone pain; loose teeth; spongy and purplish gums that are prone to bleeding; bulging eyes; scaly, dry, and brownish skin; dry hair that breaks; edema; petechiae; and easy bruising	Total plasma vitamin C (sum of AA and DHAA) or AA.	Oral: 200–500 mg daily ǂ
			HPN and long-term PN: 100–200 mg/day
***Fat-soluble vitamins***			***Doses (all values per day)***
Vitamin A (DRI: 700–900 µg/day)	Night blindness, Bitot spots, foamy appearance on the conjunctiva, xerophthalmia, increased susceptibility to infections, and impairment of the intestinal immune and barrier function	Serum retinol	Oral: 5000–50,000 IU daily #
			HPN and long-term PN: 800–1100 µg/day
Vitamin D (DRI: 15–20 µg/day)	Osteomalacia and nutritional rickets; increased susceptibility to infections	Serum 25-hydroxyvitamin D (25(OH)D)	<12 ng/mL: oral: 50,000 IU# once weekly (or calcitriol 0.25–2 mg daily), followed by maintenance: ○ 12–20 ng/mL: ▪ 800–1000 IU/day ○ 20–30 ng/mL: ▪ 600–800 IU/day
			HPN and long-term PN: 200 IU/5 µg/day
Vitamin E (DRI: 15 mg/day)	Neurological symptoms (balance and coordination disorders and peripheral neuropathy) and muscle weakness	Serum alpha-tocopherol	Oral: 400 IU up to 3 times daily
			HPN and long-term PN: 9–10 mg/day
Vitamin K (DRI: 90–120 µg/day)	Prolongation of prothrombin time with impaired clotting or bleeding, poor bone development, osteoporosis, and increased cardiovascular disease	Combination of biomarkers and dietary intake	Oral: 2.5 to 10 mg twice weekly to daily, or 10-mg single dose #; can be repeated 48–72 h later
			HPN & long-term PN: 150 µg/day, usually provided by lipid emulsions
***Trace mineral***			***Doses (all values per day)***
Iron (DRI: 8 mg/day. 18 mg/day for female 19–50 years old)	Microcytic anemia	Serum ferritin, sideremia, and transferrin saturation (%)	Oral: 100–200 mg once daily or every other day # ǂ
			HPN and long-term PN: 1.1 mg/day
Copper (DRI: 1.1–2 mg/day)	Microcytic anemia, neutropenia, osteoporosis, hair depigmentation, cardiac arrhythmias, delayed wound healing, and myeloneuropathy	Serum copper	Oral: 2 mg of elemental copper daily ǂ Higher doses may be needed
			HPN and long-term PN: 0.3–0.5 mg/day-
Chromium (DRI: 20–35 µg/day)	Hyperglycemia, insulin resistance, elevated plasma fatty acids, weight loss, and peripheral neuropathy	Serum chromium	Oral: 100–200 mg up to 3 times daily
			HPN and long-term PN: 10–15 µg/day
Zinc (DRI: 8–11 mg/day)	Impairment of immune defense, reduced growth rate, alopecia, skin rash of the face, groins, hands, and feet, delayed sexual development and bone maturation, impaired wound healing, diarrhea, and blunting of taste and smell	Serum zinc	Oral: 50 mg elemental zinc (once or twice daily) Dietary sources such as oysters and mussels can also be considered
			HPN and long-term PN: 3–5 mg/day
Selenium (DRI: 55 µg/day)	Cardiomyopathy, skeletal muscle myopathy, and skin and nail impact	Serum selenium	Oral: 100–200 mg daily
			HPN and long-term PN: 60–100 µg/day

* The doses shown in the table allow for the daily requirements to be covered. A dose adjustment would be necessary for severe deficiencies of any micronutrient that has been referred to (see Refs. [108,109,110]). # IM: administration also available; ǂ IV: administration also available HPN: home parenteral nutrition; IM: intramuscular; IV: intravenous; DRI: dietary reference intake; AI: adequate intake; DFE: dietary folate equivalent; ThDP: thiamine diphosphate; RBCs: red blood cells; NAD: nicotinamide adenine dinucleotide; PLP: plasma pyridoxal phosphate; holo-TC: holo-transcobalamin; MMA: methylmalonic acid; AA: ascorbic acid; and DHAA: dehydroascorbic acid.

**Table 9:** Clinical conditions that should be considered for the indication of ITx in patients with SBS and IF (adapted from references [91,177,178]).

Clinical Condition	Criteria	Comments
IAFLD	Forthcoming (total bilirubin above 3–6 mg/dL (54–108 μmol/L), progressive thrombocytopenia, and progressive splenomegaly) or overt liver failure (portal hypertension, hepatosplenomegaly, hepatic fibrosis, or cirrhosis because of IFALD).	Liver biopsy is the gold standard test to identify the stage of liver disease, the timing of transplantation, and the type of transplant required (isolated ITx or combined liver–ITx) It has been suggested that patients with METAVIR stage II fibrosis (perisinusoidal and portal/periportal fibrosis) should be considered for an isolated ITx, whereas those with stage III (bringing fibrosis) or IV (cirrhosis) should be considered for LITx.
	Esophageal varices, ascites, and impaired synthetic function are not always present.	
Central venous catheter-related thrombosis (CRVT)	Thrombosis of two or more central veins (loss of right and left internal jugular vein, right and left subclavian vein, or right and left femoral vein).	CRVT is a severe complication that is responsible for the loss of central venous accesses in patients on HPN and may be an indication for ITx if it affects two or more of the central venous vessels. For adults, this criterion is on a case-by-case basis.
Catheter-related bloodstream infection (CRBSI).	Frequent central line sepsis: two or more episodes per year of systemic sepsis secondary to line infections requiring hospitalization; a single episode of line-related fungemia; septic shock and/or acute respiratory distress syndrome.	Children: two admissions to an intensive care unit because of cardiorespiratory failure (mechanical ventilation or inotrope infusion) due to sepsis. For adults, this criterion is on a case-by-case basis, because recurrent episodes of CRBSI have been demonstrated to not be associated with an increased risk of death.
Other indications	Refractory electrolyte changes and frequent episodes of dehydration. High risk of death attributable to underlying diseases, such as congenital mucosal disorders, ultra-short bowel syndrome (gastrostomy; duodenostomy; residual small bowel <10 cm in infants and <20 cm in adults), and invasive intra-abdominal desmoid tumors; patients with high morbidity (frequent hospitalization, narcotic dependency, and inability to function (i.e., pseudo-obstruction; high output stoma)) or a low acceptance of long-term PN, especially in young patients.	

IAFLD: intestinal failure-associated liver disease (previously referred to as parenteral nutrition-associated liver disease, or PNALD). ITx: intestinal transplant. LITx: combined intestinal and liver transplantation. HPN: home parenteral nutrition. CRBSI: catheter-related bloodstream infection.

## Data Availability

Not applicable.

## Abbreviations

The following abbreviations were used in this manuscript: AA: ascorbic acid; AGA: American Gastroenterological Association; AI: adequate intake; ASPEN: American Association for Parenteral and Enteral Nutrition; CNS: central nervous system; DFE: dietary folate equivalent; DHAA: dehydroascorbic acid; DNA: deoxyribonucleic acid; DRI: dietary reference intake; EN: enteral nutrition; ESPEN: European Society of Nutrition and Metabolism; GH: growth hormone; GI: gastrointestinal; GLN: glutamine; holo-TC: holo-transcobalamin; HRQL: health-related quality of life; HPN: home parenteral nutrition; IBD: inflammatory bowel disease; IF: intestinal failure; IFALD: intestinal failure-associated liver disease; IM: intramuscular; ISCs: intestinal stem cells; ITx: intestinal transplantation; IV: intravenous. LAR: low anterior resection; LARS: low anterior resection syndrome; MMA: methylmalonic acid; NAD: nicotinamide adenine dinucleotide; NS: nutritional support; PLP: plasma pyridoxal phosphate; PN: parenteral nutrition; QOL: quality of life; ORSs: oral rehydration solutions; RBCs: red blood cells; RD: registered dietitian; SBS: short bowel syndrome; SCFAs: short-chain fatty acids; SIBO: small intestine bacterial overgrowth; TESIs: tissue-engineered small intestines; ThDP: thiamine diphosphate; and UDCA: ursodeoxycholic acid.
